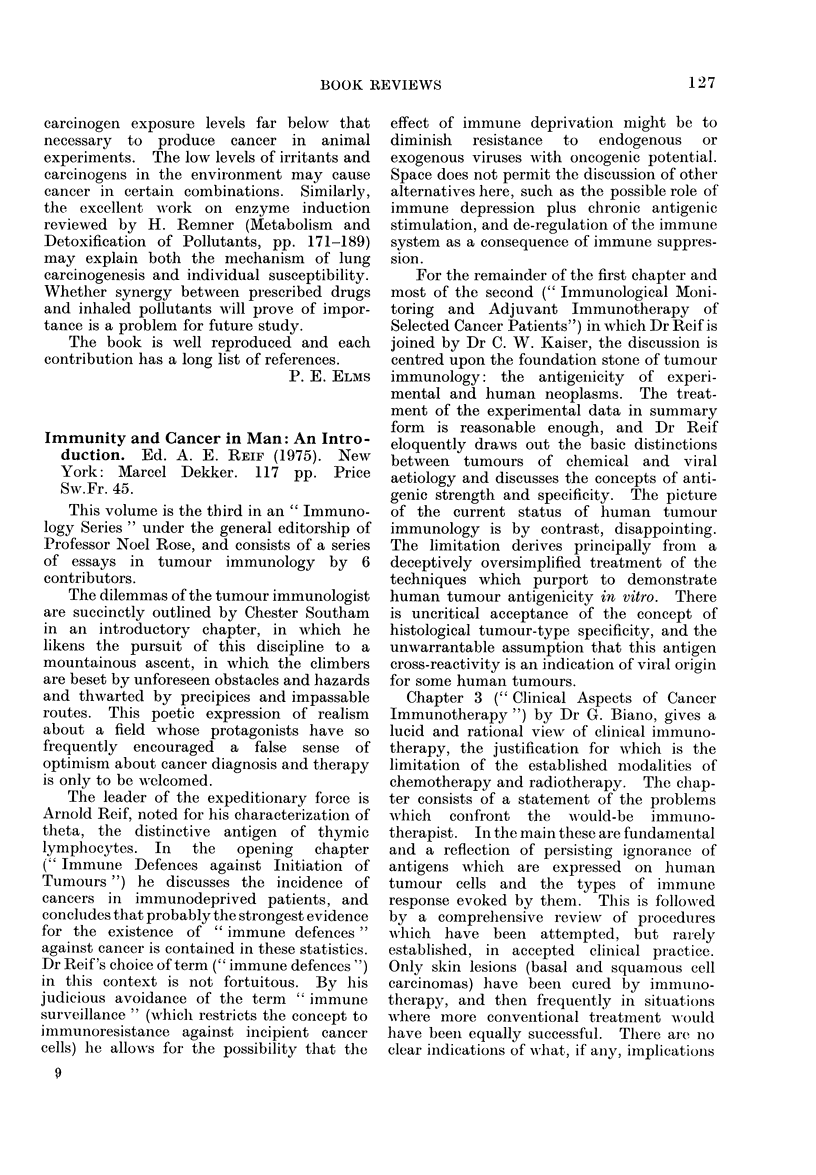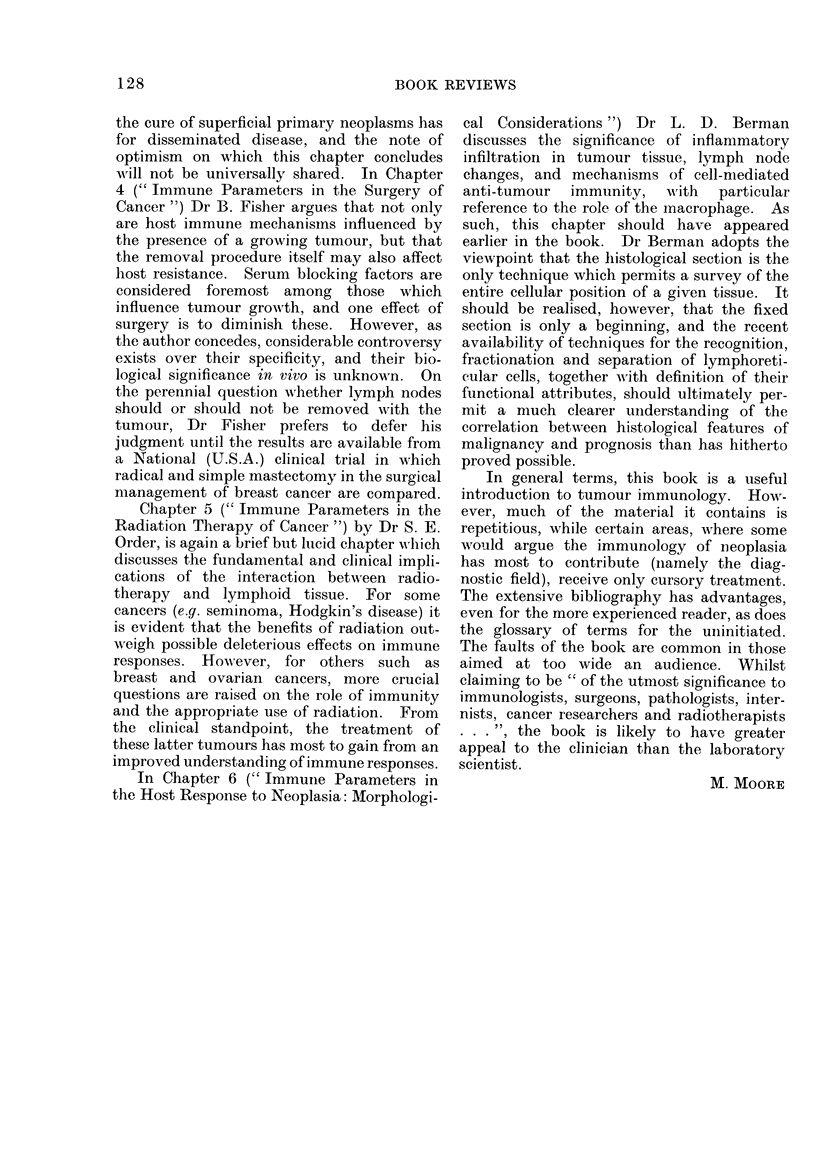# Immunity and Cancer in Man: An Introduction

**Published:** 1977-01

**Authors:** M. Moore


**Immunity and Cancer in Man: An Introduction.** Ed. A. E. Reif (1975). New York: Marcel Dekker. 117 pp. Price Sw.Fr. 45.

This volume is the third in an " Immunology Series " under the general editorship of Professor Noel Rose, and consists of a series of essays in tumour immunology by 6 contributors.

The dilemmas of the tumour immunologist are succinctly outlined by Chester Southam in an introductory chapter, in which he likens the pursuit of this discipline to a mountainous ascent, in which the climbers are beset by unforeseen obstacles and hazards and thwarted by precipices and impassable routes. This poetic expression of realism about a field whose protagonists have so frequently encouraged a false sense of optimism about cancer diagnosis and therapy is only to be welcomed.

The leader of the expeditionary force is Arnold Reif, noted for his characterization of theta, the distinctive antigen of thymic lymphocytes. In the opening chapter (" Immune Defences against Initiation of Tumours ") he discusses the incidence of cancers in immunodeprived patients, and concludes that probably the strongest evidence for the existence of " immune defences " against cancer is contained in these statistics. Dr Reif's choice of term (" immune defences ") in this context is not fortuitous. By his judicious avoidance of the term " immune surveillance " (which restricts the concept to immunoresistance against incipient cancer cells) he allows for the possibility that the effect of immune deprivation might be to diminish resistance to endogenous or exogenous viruses with oncogenic potential. Space does not permit the discussion of other alternatives here, such as the possible role of immune depression plus chronic antigenic stimulation, and de-regulation of the immune system as a consequence of immune suppression.

For the remainder of the first chapter and most of the second (" Immunological Monitoring and Adjuvant Immunotherapy of Selected Cancer Patients") in which Dr Reif is joined by Dr C. W. Kaiser, the discussion is centred upon the foundation stone of tumour immunology: the antigenicity of experimental and human neoplasms. The treatment of the experimental data in summary form is reasonable enough, and Dr Reif eloquently draws out the basic distinctions between tumours of chemical and viral aetiology and discusses the concepts of antigenic strength and specificity. The picture of the current status of human tumour immunology is by contrast, disappointing. The limitation derives principally from a deceptively oversimplified treatment of the techniques which purport to demonstrate human tumour antigenicity *in vitro*. There is uncritical acceptance of the concept of histological tumour-type specificity, and the unwarrantable assumption that this antigen cross-reactivity is an indication of viral origin for some human tumours.

Chapter 3 (" Clinical Aspects of Cancer Immunotherapy ") by Dr G. Biano, gives a lucid and rational view of clinical immunotherapy, the justification for which is the limitation of the established modalities of chemotherapy and radiotherapy. The chapter consists of a statement of the problems which confront the would-be immunotherapist. In the main these are fundamental and a reflection of persisting ignorance of antigens which are expressed on human tumour cells and the types of immune response evoked by them. This is followed by a comprehensive review of procedures which have been attempted, but rarely established, in accepted clinical practice. Only skin lesions (basal and squamous cell carcinomas) have been cured by immunotherapy, and then frequently in situations where more conventional treatment would have been equally successful. There are no clear indications of what, if any, implications the cure of superficial primary neoplasms has for disseminated disease, and the note of optimism on which this chapter concludes will not be universally shared. In Chapter 4 (" Immune Parameters in the Surgery of Cancer ") Dr B. Fisher argues that not only are host immune mechanisms influenced by the presence of a growing tumour, but that the removal procedure itself may also affect host resistance. Serum blocking factors are considered foremost among those which influence tumour growth, and one effect of surgery is to diminish these. However, as the author concedes, considerable controversy exists over their specificity, and their biological significance *in vivo* is unknown. On the perennial question whether lymph nodes should or should not be removed with the tumour, Dr Fisher prefers to defer his judgment until the results are available from a National (U.S.A.) clinical trial in which radical and simple mastectomy in the surgical management of breast cancer are compared.

Chapter 5 (" Immune Parameters in the Radiation Therapy of Cancer ") by Dr S. E. Order, is again a brief but lucid chapter which discusses the fundamental and clinical implications of the interaction between radiotherapy and lymphoid tissue. For some cancers (*e.g.* seminoma, Hodgkin's disease) it is evident that the benefits of radiation outweigh possible deleterious effects on immune responses. However, for others such as breast and ovarian cancers, more crucial questions are raised on the role of immunity and the appropriate use of radiation. From the clinical standpoint, the treatment of these latter tumours has most to gain from an improved understanding of immune responses.

In Chapter 6 (" Immune Parameters in the Host Response to Neoplasia: Morphological Considerations ") Dr L. D. Berman discusses the significance of inflammatory infiltration in tumour tissue, lymph node changes, and mechanisms of cell-mediated anti-tumour immunity, with particular reference to the role of the macrophage. As such, this chapter should have appeared earlier in the book. Dr Berman adopts the viewpoint that the histological section is the only technique which permits a survey of the entire cellular position of a given tissue. It should be realised, however, that the fixed section is only a beginning, and the recent availability of techniques for the recognition, fractionation and separation of lymphoreticular cells, together with definition of their functional attributes, should ultimately permit a much clearer understanding of the correlation between histological features of malignancy and prognosis than has hitherto proved possible.

In general terms, this book is a useful introduction to tumour immunology. However, much of the material it contains is repetitious, while certain areas, where some would argue the immunology of neoplasia has most to contribute (namely the diagnostic field), receive only cursory treatment. The extensive bibliography has advantages, even for the more experienced reader, as does the glossary of terms for the uninitiated. The faults of the book are common in those aimed at too wide an audience. Whilst claiming to be " of the utmost significance to immunologists, surgeons, pathologists, internists, cancer researchers and radiotherapists . . . ", the book is likely to have greater appeal to the clinician than the laboratory scientist.

M. Moore